# Tailoring and Fabricating Temperature-Stable ZnNb_2_O_6_-Ca_0.5_Sr_0.5_TiO_3_ Composite Ceramics for Next-Generation Microwave Components

**DOI:** 10.3390/ma18245572

**Published:** 2025-12-11

**Authors:** Haodong Wang, Chuying Chen, Xiuli Fu, Zhijian Peng

**Affiliations:** 1 School of Engineering and Technology, China University of Geosciences, Beijing 100083, China; hdwang@cugb.edu.cn; 2 School of Science, China University of Geosciences, Beijing 100083, China; 4122153061@stu.xjtu.edu.cn; 3 School of Science, Beijing University of Posts and Telecommunications, Beijing 100876, China; xiulfu@bupt.edu.cn

**Keywords:** composite ceramics, ZnNb_2_O_6_, Ca_0.5_Sr_0.5_TiO_3_, sintering, microwave dielectric properties

## Abstract

**Highlights:**

**What are the main findings?**

- Novel ZnNb_2_O_6_-Ca_0.5_Sr_0.5_TiO_3_ composite ceramics were designed.
- Compositional effect was systematically investigated.
- Optimum sintering system was established.
- Temperature-stable microwave ceramics were obtained.

**What are the implications of the main findings?**

- Theoretical guide and material support for designing and fabricating high-performance thermally stable microwave dielectric ceramics for future communication technologies.

**Abstract:**

ZnNb_2_O_6_-based microwave dielectric ceramics have attracted considerable attention due to their high quality factor (Q × f) and low sintering temperature, but their application was limited by poor temperature stability with a large negative temperature coefficient of resonant frequency (τ_f_). Herein, novel (1 − x)ZnNb_2_O_6−x_Ca_0.5_Sr_0.5_TiO_3_ (x = 0.05–0.125) composite ceramics were designed and fabricated. The used ZnNb_2_O_6_ and Ca_0.5_Sr_0.5_TiO_3_ were synthesized through solid-phase reaction by using stoichiometric metal oxides or carbonates as the raw materials at 650 and 1100 °C, respectively. The composite ceramics were prepared by solid-state sintering, and the sintering parameters were optimized at 1175 °C for 4 h by visual high-temperature deformation analysis. A focus was paid on the temperature stability and compositional effects of Ca_0.5_Sr_0.5_TiO_3_ of the obtained composited ceramics. As the Ca_0.5_Sr_0.5_TiO_3_ content increases, the dielectric constant (ε_r_) and Q × f gradually decrease, while τ_f_ shifts toward positive values. At x = 0.075, the composite ceramics sintered at 1175 °C for 4 h exhibit near-zero τ_f_ (−8.99 ppm/°C), coupled with ε_r_ = 23.23 and Q × f = 21,686 GHz. This study provides theoretical guide and material support for designing and fabricating various high-performance thermally stable microwave dielectric ceramics for 5G communication devices and future communication technologies.

## 1. Introduction

The rapid development of 5G communication technology has led to a significant increase in global demand for high-performance microwave components [1,2,3]. As essential materials in modern communication systems, microwave dielectric ceramics are widely used in the fabrication of resonators, filters, substrates, antennas, waveguide circuits and so on [4,5,6,7,8]. These components form the foundation of wireless communication systems, enabling higher data transmission rates, broader bandwidths, and improved signal integrity. However, the ongoing evolution of 5G and the impending arrival of 6G technologies necessitate the development of microwave dielectric ceramics with superior performance to meet increasingly stringent requirements [9,10,11].

To meet these demands, high-performance microwave dielectric ceramics must satisfy three critical criteria: (i) an appropriate relative dielectric constant (ε*ᵣ*) for device miniaturization; (ii) a high quality factor (Q × f) or low dielectric loss to ensure energy efficiency; and (iii) a near-zero temperature coefficient of resonant frequency (τ*_f_*) to guarantee stability under varying operating conditions [12,13,14]. However, single-phase ceramic materials often face inherent limitations in simultaneously achieving these properties. As a result, researchers have turned to composite strategies to optimize the overall performance of microwave dielectric ceramics [15,16].

In recent years, niobate-based microwave dielectric ceramics have attracted considerable attention due to their excellent dielectric properties [17,18,19]. Among them, ZnNb_2_O_6_ ceramics have become a research focus due to their low dielectric constant (ε*ᵣ* ≈ 25), high quality factor (Q × f ≈ 83,700 GHz), and low sintering temperature (1150 °C) [20,21]. However, ZnNb_2_O_6_ ceramics exhibit a large negative τ*_f_* (−56.1 ppm/°C), resulting in poor temperature stability and limiting their practical applications [18,19,20]. Studies on ion substitution, such as Mg^2+^ or Ni^2+^ for Zn^2+^, have improved τ_f_ but introduced challenges such as increased sintering temperatures or dielectric losses [22,23,24].

To address this issue, composite strategies combining ZnNb_2_O_6_ with materials of positive τ*_f_* values have been proposed [19,25,26,27,28,29,30]. Among such materials, Ca_0.5_Sr_0.5_TiO_3_, a perovskite-structured ceramic with ε*ᵣ* ≈ 236, Q × f ≈ 4100 GHz and τ*_f_* ≈ +1230 ppm/°C, ushers in an ideal candidate for ZnNb_2_O_6_ [31,32,33]. Its structural compatibility with ZnNb_2_O_6_ facilitates the formation of composite ceramics, minimizing secondary phases and optimizing dielectric performance.

Therefore, this study designed and fabricated a series of novel (1 − x)ZnNb_2_O_6−x_ Ca_0.5_Sr_0.5_TiO_3_ (x = 0.05–0.125) materials, aiming to search for a kind of composite ceramic with near-zero τ*_f_* but acceptable Q × f. The used ZnNb_2_O_6_ and Ca_0.5_Sr_0.5_TiO_3_ were synthesized through solid-phase reaction by using stoichiometric metal oxides or carbonates as the raw materials. The composite ceramics were prepared by solid-state sintering, and the sintering parameters were optimized by using visual high-temperature deformation analysis. The effects of Ca_0.5_Sr_0.5_TiO_3_ content on the phase composition, microstructure, sintering behavior, and microwave dielectric properties of the obtained composite ceramics were systematically investigated. By optimizing the composition and sintering process, ZnNb_2_O_6_-Ca_0.5_Sr_0.5_TiO_3_ composite ceramics with excellent dielectric properties and high temperature stability were obtained. With x = 0.075 after sintering at 1175 °C for 4 h, the obtained composite ceramics exhibit near-zero τ_f_ (−8.99 ppm/°C), ε_r_ = 23.23 and Q × f = 21,686 GHz. This study provides theoretical guide and material support for designing and fabricating various high-performance microwave dielectric ceramics for 5G communication devices and future communication technologies.

## 2. Experimental

### 2.1. Sample Fabrication

The proposed (1 − x)ZnNb_2_O_6−x_Ca_0.5_Sr_0.5_TiO_3_ (x = 0.05–0.125) composite ceramics were prepared using the conventional solid-phase reaction and solid-state sintering method. Typically, high-purity raw materials, including Nb_2_O_5_ (99.99%), ZnO (99.99%), CaCO_3_ (99.99%), SrCO_3_ (99.99%), and TiO_2_ (99.99%), were weighed according to their stoichiometric ratios. The raw materials were mixed with anhydrous ethanol and ZrO_2_ grinding balls in a planetary ball mill. Specifically, Nb_2_O_5_ and ZnO were mixed and ball-milled for 6 h, while CaCO_3_, SrCO_3_, and TiO_2_ were mixed and ball-milled for 8 h. After ball milling, the slurries were quickly transferred to an oven and dried at 105 °C for 8 h. The dried mixtures were then ground into fine powders using an agate mortar. The powders were calcined in a muffle furnace to obtain ZnNb_2_O_6_ and Ca_0.5_Sr_0.5_TiO_3_. ZnNb_2_O_6_ was synthesized by calcining the Nb_2_O_5_-ZnO mixture at 650 °C for 4 h, while Ca_0.5_Sr_0.5_TiO_3_ was synthesized by calcining the CaCO_3_-SrCO_3_-TiO_2_ mixture at 1100 °C for 4 h. After crashing and grinding, the resultant ZnNb_2_O_6_ and Ca_0.5_Sr_0.5_TiO_3_ powders were weighed according to the designed ratios of composite ceramics, mixed, and ball-milled again for 6 h. The obtained slurries were dried at 105 °C for 8 h, and then the resultant powder chunks were ground and sieved to obtain homogeneous ceramic powders. To improve the formability of the powders, 0.5 wt.% polyvinyl alcohol (PVA) was added as a binder for the granulation of the composite powders. Afterwards, the granulated powders were pressed into pellets (ϕ5 × 1 mm, ϕ6 × 1 mm, and ϕ12 × 5 mm) using a hydraulic press under a pressure of 4 MPa for 90 s.

To determine the optimal sintering temperature, the green pellets (ϕ5 × 1 mm) were subjected to high-temperature deformation analysis using a visual high-temperature deformation analyzer (TA-16A01, Tianjin Zhonghuan Experimental Furnace Co., Ltd., Tianjin, China). The samples were heated at a rate of 5 °C/min up to 1350 and 1300 °C, respectively, and the data were analyzed to determine the optimal sintering temperature. The final sintering process was carried out in a muffle furnace. The samples were heated at a rate of 5 °C/min up to 500 °C, holding there for 30 min to remove the binder, and then heated up to the optimal sintering temperature (1175 °C), soaking for 4 h before cooling naturally to room temperature.

### 2.2. Material Characterization

The apparent density of the sintered ceramics was measured using the Archimedes method, following the ISO 18754 standard [34]. The relative density was calculated as the ratio of the apparent density to the theoretical density, expressed as a percentage. The phase composition of the ceramics was analyzed using an X-ray diffractometer (XRD, D/max-RB, Rigaku, Tokyo, Japan) with Cu-Kα radiation (λ = 1.5418 Å). The XRD patterns were collected in continuous scanning mode at a rate of 5°/min over a 2θ range of 10–80°. The microstructure of the ceramics was observed using a scanning electron microscope (SEM, Meilin Compact, Zeiss, Oberkochen, Germany). The average grain size and distribution were statistically analyzed from the SEM images using Nano Measurer software. The elemental composition and distribution were analyzed using an energy-dispersive X-ray spectrometer (EDX, Inca X-Max 80T, Oxford, UK).

The microwave dielectric properties, including the dielectric constant (ε*ᵣ*), quality factor (Q × f), and temperature coefficient of resonant frequency (τ*_f_*), were measured using a vector network analyzer (E5063A, Keysight, San Diego, CA, USA) in TE01δ mode. The resonant frequency temperature coefficient (τ*_f_*) was calculated using the following formula [33,35]:

(1) $$\tau_{f}=\frac{f_{t}-f_{0}}{f_{0}\left(t-t_{0}\right)}$$

where *f_t_* and *f*_0_ are the resonant frequencies at 80 °C and room temperature, respectively.

## 3. Results and Discussion

### 3.1. Synthesis and Sintering Behavior

**Figure 1:**
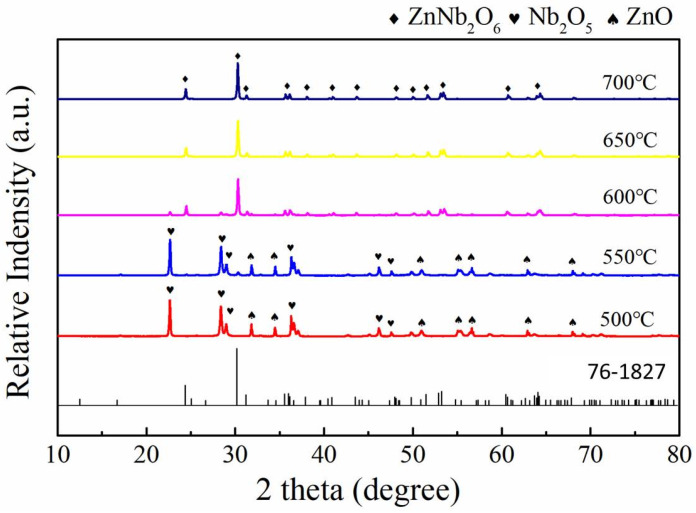
XRD patterns of the products obtained after calcining Nb_2_O_5_ and ZnO mixed powders in a molar ratio of 1:1 at 500, 550, 600, 650 and 700 °C for 4 h, respectively.

**Figure 2:**
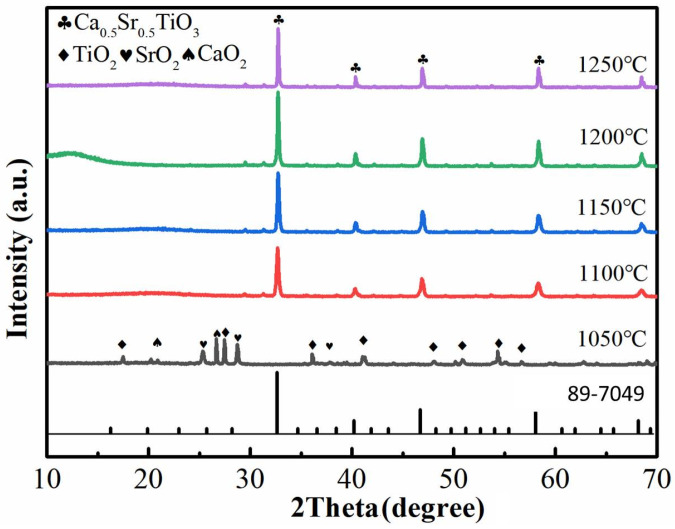
XRD patterns of the products obtained after CaCO_3_, SrCO_3_, TiO_2_ mixed powders in a molar ratio of 1:1:2 were calcined at 1050, 1100, 1150, 1200 and 1250 °C for 4 h, respectively.

The synthesis of ZnNb_2_O_6_ and Ca_0.5_Sr_0.5_TiO_3_ raw powders via the solid-phase reaction method was confirmed by XRD analysis to determine the lowest synthesis temperature. High-purity Nb_2_O_5_ and ZnO were mixed in a molar ratio of 1:1, ball-milled with ethanol for 6 h, dried, and calcined at temperatures ranging from 500 to 700 °C for 4 h to obtain ZnNb_2_O_6_ [29,36]. As shown in Figure 1, the diffraction peaks of ZnNb_2_O_6_ were clearly observed at 600 °C, although a small amount of Nb_2_O_5_ still remained. At 650 °C, pure-phase ZnNb_2_O_6_ was obtained, and further increases in temperature did not alter the phase composition. Therefore, 650 °C was selected as the calcination temperature for ZnNb_2_O_6_ from Nb_2_O_5_ and ZnO mixed powders. Similarly, CaCO_3_, SrCO_3_, and TiO_2_ were mixed in a molar ratio of 1:1:2, ball-milled for 8 h, dried, and calcined at temperatures ranging from 1050 to 1250 °C for 4 h to obtain Ca_0.5_Sr_0.5_TiO_3_ [33,37]. From Figure 2, it can be observed that pure-phase Ca_0.5_Sr_0.5_TiO_3_ was obtained at 1100 °C, and no phase changes occurred at higher temperatures. Thus, 1100 °C was chosen as the calcination temperature for Ca_0.5_Sr_0.5_TiO_3_ from CaCO_3_, SrCO_3_, TiO_2_ mixed powders.

**Figure 3:**
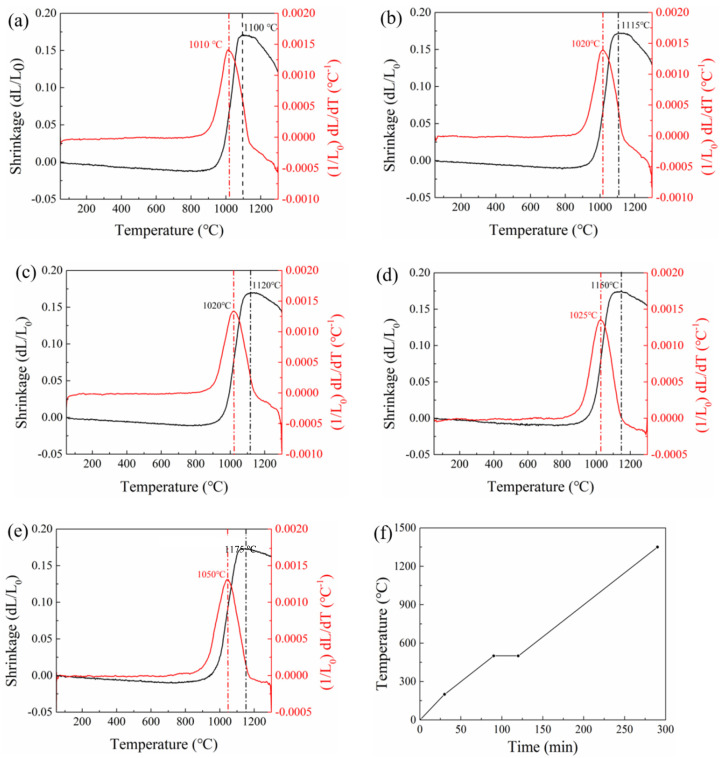
Shrinkage behavior of (1−x)ZnNb_2_O_6−x_Ca_0.5_Sr_0.5_TiO_3_ ceramics as a function of temperature (**a**–**e**) and time (**f**): (**a**) x = 0.025, (**b**) x = 0.05, (**c**) x = 0.075, (**d**) x = 0.1, (**e**) x = 0.125. (**f**) Heating process for the deformation analysis of the present ZnNb_2_O_6_-Ca_0.5_Sr_0.5_TiO_3_ composite ceramics.

To determine the optimal sintering temperature for the proposed (1 − x)ZnNb_2_O_6−x_Ca_0.5_Sr_0.5_TiO_3_ (x = 0.05–0.125) composite ceramics, their green pellets (ϕ5 × 1 mm) were subjected to high-temperature deformation analysis. The collected shrinkage curves are shown in Figure 3a–e, for which the samples were heated according to the process as illustrated in Figure 3f. It can be seen from Figure 3a–e that the sintering temperature for the composite ceramics increased with higher Ca_0.5_Sr_0.5_TiO_3_ content, indicating that excessive Ca_0.5_Sr_0.5_TiO_3_ addition hindered their densification. Roughly at 1175 °C (see Figure 3e), the designed composition of composite ceramics with the highest Ca_0.5_Sr_0.5_TiO_3_ content can be sintered densely for 4 h. Based on these results, the optimal sintering parameters for all the proposed composite ceramics were determined to be 1175 °C for 4 h.

### 3.2. Composition and Microstructure

**Figure 4:**
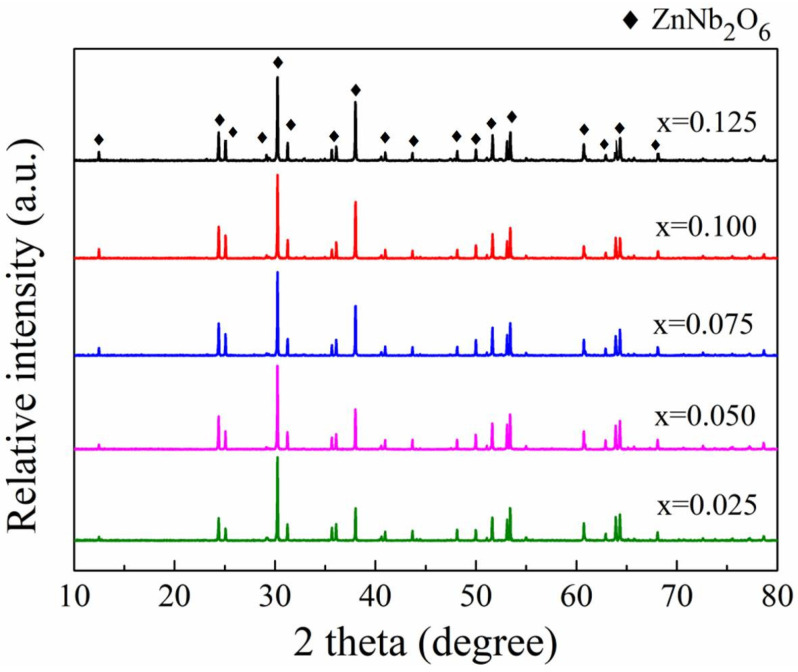
XRD patterns of the (1 − x)ZnNb_2_O_6−x_Ca_0.5_Sr_0.5_TiO_3_ ceramics obtained by sintering at 1175 °C for 4 h.

The XRD patterns of the obtained (1 − x)ZnNb_2_O_6−x_Ca_0.5_Sr_0.5_TiO_3_ (x = 0.05–0.125) ceramics sintered at 1175 °C for 4 h are shown in Figure 4. For all the samples, the primary phase was identified as ZnNb_2_O_6_, while the peaks of Ca_0.5_Sr_0.5_TiO_3_ phase were not detected due to its low content. This information indicates that the composition of the obtained ceramics is desirable as designed without extra reaction during sintering.

**Figure 5:**
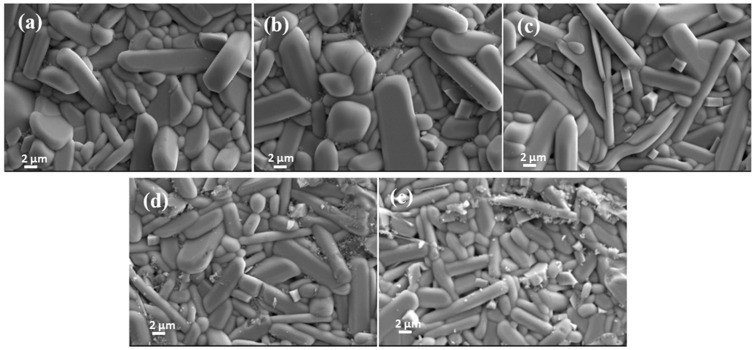
SEM images on the polished and thermally etched surfaces of (1 − x)ZnNb_2_O_6−x_Ca_0.5_Sr_0.5_TiO_3_ ceramics obtained by sintering at 1175 °C for 4 h: (**a**) x = 0.025, (**b**) x = 0.05, (**c**) x = 0.075, (**d**) x = 0.1, (**e**) x = 0.125.

**Figure 6:**
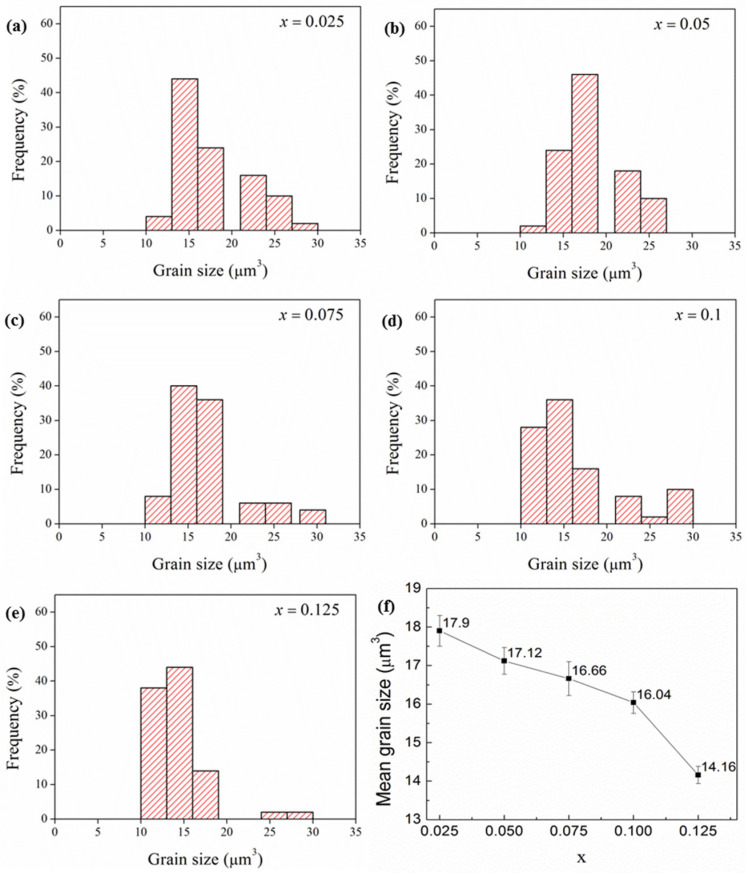
Distribution diagram of grain sizes (μm^3^) in the obtained (1 − x)ZnNb_2_O_6−x_Ca_0.5_Sr_0.5_TiO_3_ ceramics: (**a**) x = 0.025, (**b**) x = 0.05, (**c**) x = 0.075, (**d**) x = 0.1, (**e**) x = 0.125. (**f**) Average grain size changes with x.

The microstructural features on the polished and thermally etched surfaces of the obtained samples (see Figure 5) revealed that the ceramics overwhelmingly consist of rod-like grains, typical morphology of ZnNb_2_O_6_ grains, because the content of Ca_0.5_Sr_0.5_TiO_3_ was generally very small. Statistical analysis on the size distribution of the ZnNb_2_O_6_ grains (volume, μm^3^, see Figure 6) indicated that the average grain size decreased with increasing Ca_0.5_Sr_0.5_TiO_3_ content. But, with more Ca_0.5_Sr_0.5_TiO_3_ added, the size of ZnNb_2_O_6_ grains became more homogeneous. This trend is attributed to the higher sintering temperature of Ca_0.5_Sr_0.5_TiO_3_ (~1400 °C) [32,33] compared to that of ZnNb_2_O_6_ (<1020 °C) [21,24], which would impede the grain growth of ZnNb_2_O_6_ and densification of the products, while the adding amount of Ca_0.5_Sr_0.5_TiO_3_ increased. In addition, with relatively high content of Ca_0.5_Sr_0.5_TiO_3_ (for example, Figure 5d), tiny grains were gradually examined from the images, which were identified by EDX analysis as Ca_0.5_Sr_0.5_TiO_3_.

**Figure 7:**
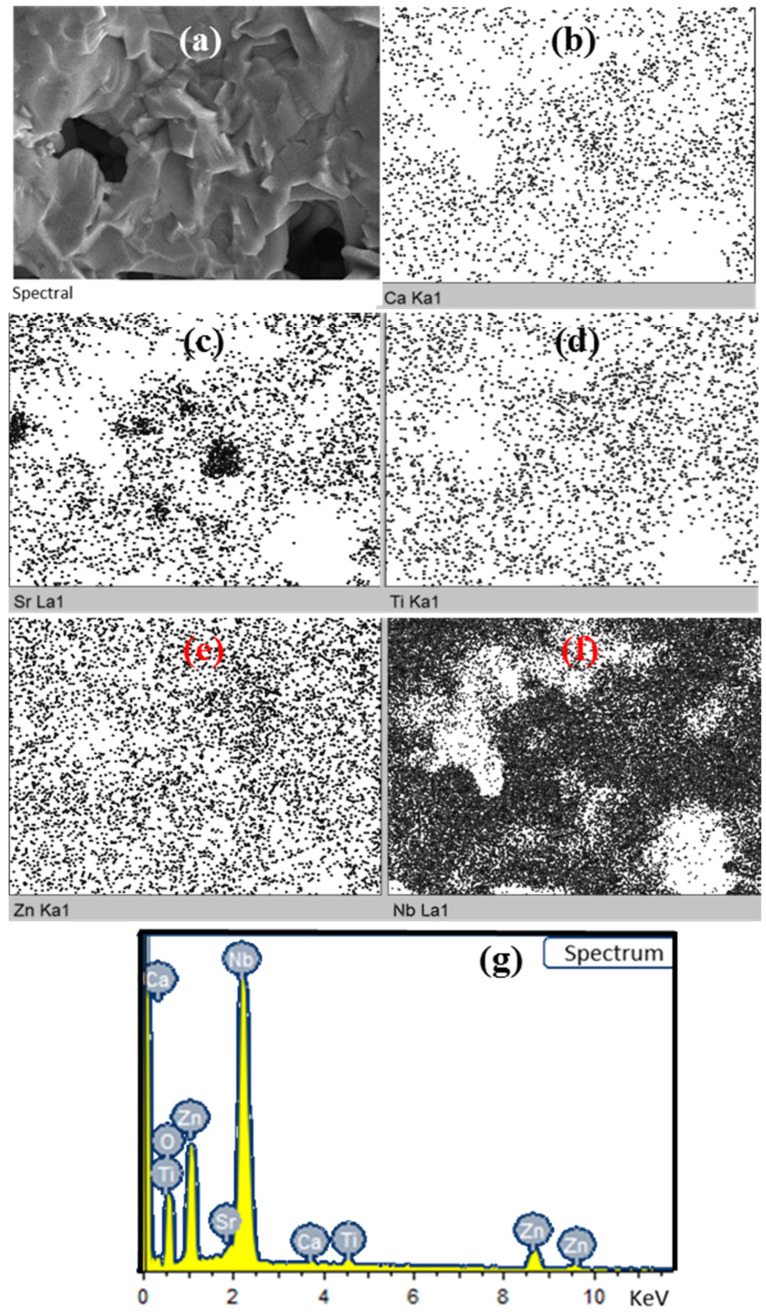
(**a**) Selected area on the fresh fracture surface of 0.925ZnNb_2_O_6_-0.075Ca_0.5_Sr_0.5_TiO_3_ ceramic sample. Corresponding elemental SEM-EDX mapping images: (**b**) Ca, (**c**) Sr, (**d**) Ti, (**e**) Zn, (**f**) Nb. And (**g**) the collected SEM-EDX spectrum.

EDX mapping analysis (see Figure 7 and Table 1) confirmed the uniform distribution of Ca, Sr, Ti, Zn and Nb elements over the samples, where the concentrations of Ca, Sr and Ti approximated to the stoichiometric ratio of Ca_0.5_Sr_0.5_TiO_3_, and the ratio of Zn and Nb presented a deviation from the stoichiometric ratio of ZnNb_2_O_6_ due to the evaporation of low boiling-point Zn^2+^ ions [19,29]. Anyway, the elemental composition together with the XRD result suggests the formation of a composite between ZnNb_2_O_6_ and Ca_0.5_Sr_0.5_TiO_3_ [38,39].

**Figure 8:**
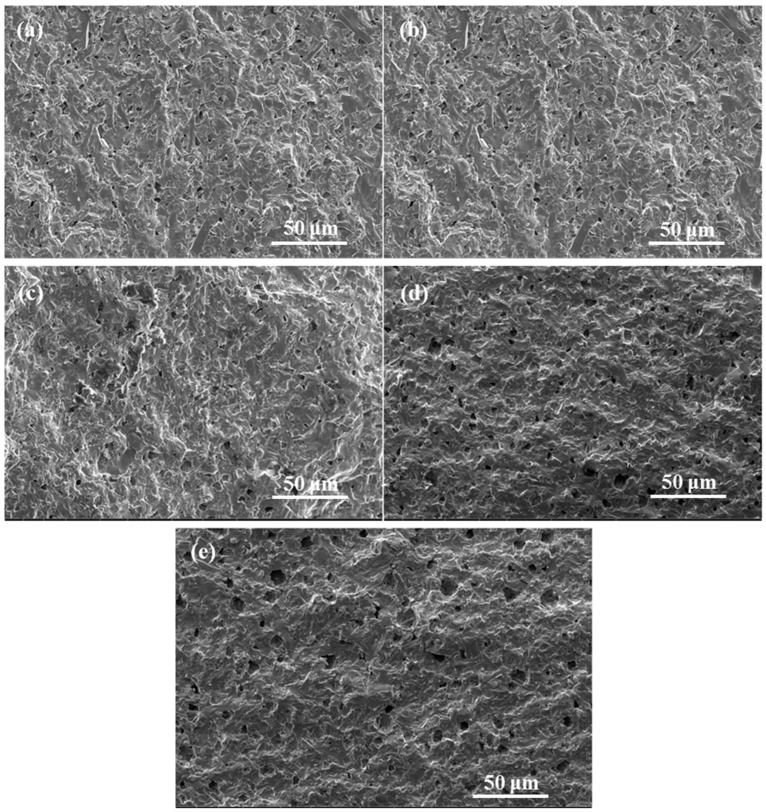
Images on the fresh fracture surface of (1 − x)ZnNb_2_O_6−x_Ca_0.5_Sr_0.5_TiO_3_ ceramics obtained by heating at 1175 °C for 4 h: (**a**) x = 0.025, (**b**) x = 0.05, (**c**) x = 0.075, (**d**) x = 0.1, (**e**) x = 0.125.

The SEM images on the fresh fracture surface of the obtained ZnNb_2_O_6_-Ca_0.5_Sr_0.5_TiO_3_ composite ceramics are shown in Figure 8. It can be clearly seen from this figure that with the increase in Ca_0.5_Sr_0.5_TiO_3_ content, the number and size of pores in the composite ceramic samples are increasing. This result confirms that during sintering, the addition of Ca_0.5_Sr_0.5_TiO_3_ would impede the densification of the designed ZnNb_2_O_6_-Ca_0.5_Sr_0.5_TiO_3_ composite ceramics. The phenomenon can be explained as follows. The sintering temperature of Ca_0.5_Sr_0.5_TiO_3_ ceramics is around 1400 °C, which is much higher than that of ZnNb_2_O_6_ (<1020 °C). And the increase in the content of high sintering temperature components is not conducive to the sintering of composite ceramics [40]. As a result, the pores cannot be removed in time during the growth of the grains, hindering the movement of the grains, finally resulting in a decrease in grain size and an increase in ceramic porosity.

**Figure 9:**
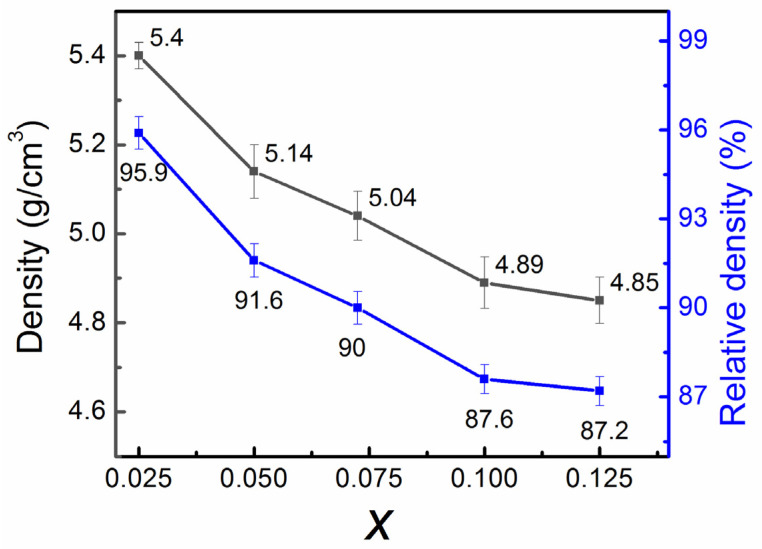
Apparent density and relative density of the obtained (1 − x)ZnNb_2_O_6−x_Ca_0.5_Sr_0.5_TiO_3_ composite ceramics as a function of x.

The apparent density and relative density of the ceramics were further measured by Archimedes method, and the results are shown in Figure 9. The apparent density of the samples decreased with increasing Ca_0.5_Sr_0.5_TiO_3_ content due to its lower density (4.7 g/cm^3^) [41] compared to that of ZnNb_2_O_6_ (5.65 g/cm^3^) [42] and decreased densification of the products. Accordingly, the relative density also decreased, primarily due to the increased porosity of the products. This result is completely consistent with the microstructure observation by SEM imaging (see Figure 8).

### 3.3. Microwave Dielectric Properties

**Figure 10:**
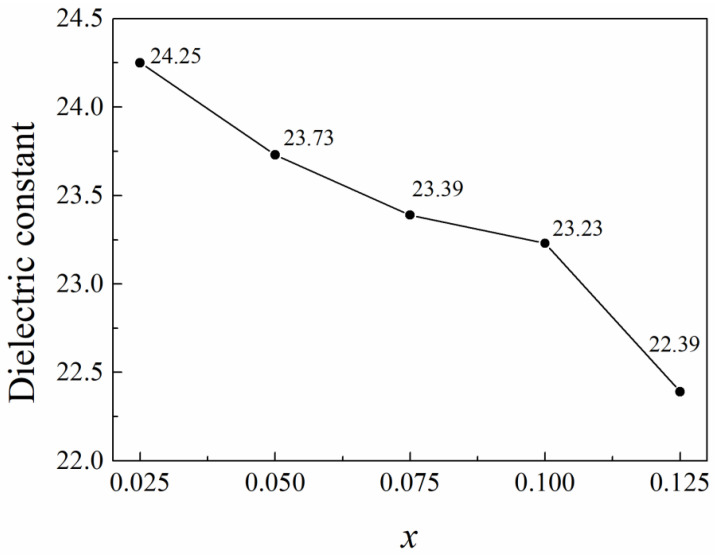
Relationship between dielectric constant (ε*ᵣ*) and x of the obtained (1 − x) ZnNb_2_O_6−x_Ca_0.5_Sr_0.5_TiO_3_ ceramics.

The dielectric constant (εᵣ) of the obtained (1 − x)ZnNb_2_O_6−x_Ca_0.5_Sr_0.5_TiO_3_ ceramics decreased with increasing Ca_0.5_Sr_0.5_TiO_3_ content (see Figure 10). Generally speaking, *ε_r_* is mainly determined by the ionic polarizabilities, second phases and pores/density in microwave dielectric ceramics [43]. For the present (1 − x)ZnNb_2_O_6−x_Ca_0.5_Sr_0.5_TiO_3_ composite ceramics, the *ε_r_* value would not be affected by ionic polarizabilities because ion substitution did not occur. Moreover, because the second phase of the added Ca_0.5_Sr_0.5_TiO_3_ has a much higher *ε_r_* (236) [33] than that of ZnNb_2_O_6_ (25) [20,21], the theoretical *ε_r_* value of the composite ceramics should increase with more Ca_0.5_Sr_0.5_TiO_3_ when the samples are of full densification. However, the actual measured *ε_r_* value of the present composite ceramics decreased when more Ca_0.5_Sr_0.5_TiO_3_ was added, indicating that the effect of Ca_0.5_Sr_0.5_TiO_3_ on the *ε_r_* value of the obtained ceramics could be neglected because the addition amount of Ca_0.5_Sr_0.5_TiO_3_ was very small. Therefore, it can be concluded that the increased porosity in the composite ceramics led to the reduction in εᵣ of the present (1 − x)ZnNb_2_O_6−x_Ca_0.5_Sr_0.5_TiO_3_ composite ceramics.

**Figure 11:**
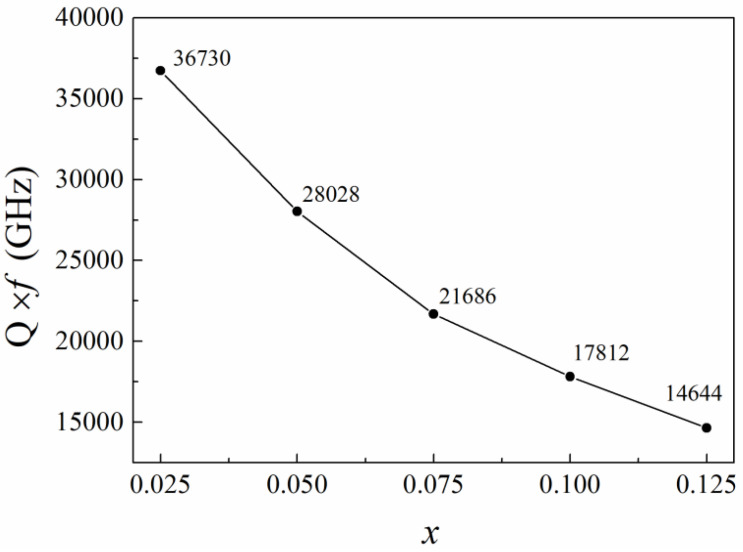
Relationship between quality factor (Q × f) and x of the obtained (1 − x)ZnNb_2_O_6−x_Ca_0.5_Sr_0.5_TiO_3_ ceramics.

The quality factor (*Q* × *f*) also decreased with higher Ca_0.5_Sr_0.5_TiO_3_ content (Figure 11). It is known that the factors influencing the *Q* × *f* value of microwave ceramics include not only the intrinsic ones mainly contributing to the lattice vibration mode but also the extrinsic ones such as the second phases in them, grains morphology and densification of the samples [43]. For the present (1 − x)ZnNb_2_O_6−x_Ca_0.5_Sr_0.5_TiO_3_ composite ceramics, the extrinsic factors clearly play a major role in the *Q* × *f* value decrease, because the dominant phase in the composite ceramics is ZnNb_2_O_6_ with an almost constant intrinsic contribution to *Q* × *f*. In other words, the decrease in *Q* × *f* value is attributed to the lower *Q* × *f* of Ca_0.5_Sr_0.5_TiO_3_ (4100 GHz) [32,33], compared to that of ZnNb_2_O_6_ (83,700 GHz) [20,21], as well as the increased dielectric losses caused by increased porosity (see Figure 9) and more grain-boundary defects with decreased grain size (see Figure 6).

**Figure 12:**
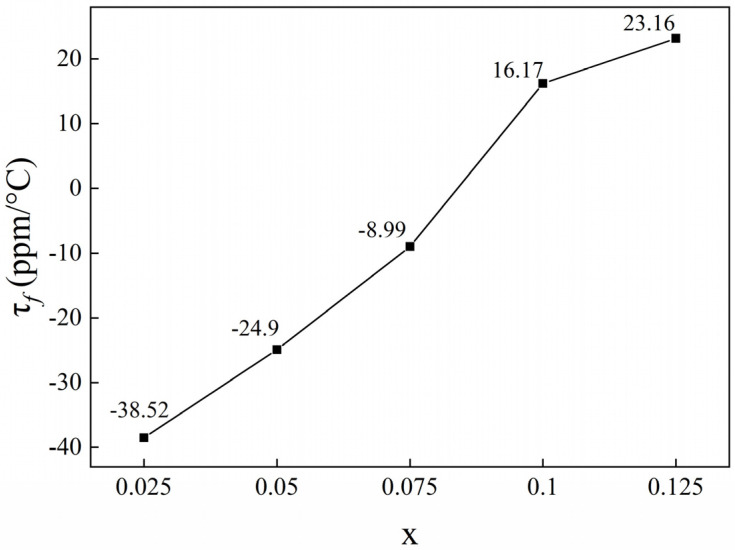
Relationship between frequency temperature coefficient (τ*_f_*) and x of the obtained (1 − x)ZnNb_2_O_6−x_Ca_0.5_Sr_0.5_TiO_3_ ceramics.

The temperature coefficient of resonant frequency (τ_f_) shifted toward positive values with increasing Ca_0.5_Sr_0.5_TiO_3_ content (Figure 12). At x = 0.075, τ_f_ reached a near-zero value (−8.99 ppm/°C), consistent with theoretical predictions. This phenomenon can be explained as follows. Generally, the *τ_f_* value of two-phase microwave dielectric composite ceramics was determined by the matrix and second phases in the samples, obeying the following mixing rule [43]:

(2) $$\tau_{ƒ}=\;\nu_{1}\tau_{ƒ1}+\nu_{2}\tau_{ƒ2},$$

where *v*_1_ and *v*_2_ represent the volume fraction of the components, and *τ_f_*_1_ and *τ_f_*_2_ are their *τ_f_* value, respectively. For the present (1 − x)ZnNb_2_O_6−x_Ca_0.5_Sr_0.5_TiO_3_ composite ceramics, the *τ_f_* value of matrix ZnNb_2_O_6_ is −56.1 ppm/°C, while that of the second phase Ca_0.5_Sr_0.5_TiO_3_ is +1230 ppm/°C. After adding Ca_0.5_Sr_0.5_TiO_3_, the *τ_f_* value of the composite ceramics would be adjusted from negative to positive effectively.

To make the merits of the obtained ZnNb_2_O_6_-Ca_0.5_Sr_0.5_TiO_3_ composite ceramics more clear, Table 2 compares the microwave dielectric properties of the present optimal 0.925ZnNb_2_O_6_-0.075Ca_0.5_Sr_0.5_TiO_3_ composite ceramics with those of the already-reported important ZnNb_2_O_6_-based microwave dielectric ceramics in the literature. It can be seen that the present 0.925ZnNb_2_O_6_-0.075Ca_0.5_Sr_0.5_TiO_3_ composite ceramics have a lower *ε_r_*, relatively high *Q* × *f* and near-zero *τ_f_* value, which would be a very promising candidate for 5G communication devices and future communication technologies.

## 4. Conclusions

This study successfully designed and fabricated novel (1 − x)ZnNb_2_O_6−x_ Ca_0.5_Sr_0.5_TiO_3_ (x = 0.05–0.125) composite ceramics with near-zero τ*_f_* but acceptable Q × f. The study systematically investigated the sintering behavior, microstructure, and microwave dielectric properties of the composite ceramics. The optimal sintering conditions were determined as 1175 °C for 4 h using a visual high-temperature deformation analyzer, ensuring full densification of all compositions. In the sintered ceramics, only the ZnNb_2_O_6_ phase was detected, while the Ca_0.5_Sr_0.5_TiO_3_ phase remained undetectable due to its low content. As the Ca_0.5_Sr_0.5_TiO_3_ content increased, the average grain size of the ceramics exhibited a decreasing trend and the relative density declined, attributed to the higher sintering temperature of Ca_0.5_Sr_0.5_TiO_3_, which impeded the grain growth and densification of ZnNb_2_O_6_-based ceramics.

With increasing Ca_0.5_Sr_0.5_TiO_3_ content, the dielectric constant (εᵣ) and quality factor (Q × f) decreased continuously, whereas the temperature coefficient of resonant frequency (τ*_f_*) shifted toward positive values. For the optimal ceramics (with x = 0.075), the τ*_f_* reached a near-zero value of −8.99 ppm/°C, accompanied by εᵣ = 23.23 and Q × f = 21,686 GHz.

These results demonstrate that adjusting the Ca_0.5_Sr_0.5_TiO_3_ content effectively optimizes the microstructure and microwave dielectric performance of ZnNb_2_O_6_-Ca_0.5_Sr_0.5_TiO_3_ composite ceramics, rendering them suitable for high-performance dielectric applications. This study provides the theoretical and material support for designing and fabricating various high-performance microwave dielectric ceramics for 5G communication devices and future communication technologies.

## Figures and Tables

**Table 1:** The elemental compositions of typical samples measured by SEM-EDX.

Elements	Ca	Sr	Ti	Zn	Nb	O
Composition (wt%)	0.59	1.39	1.66	19.72	53.06	23.57

**Table 2:** Comparison on the microwave dielectric properties of the ZnNb_2_O_6_-based microwave dielectric ceramics between the present study and those in the literature.

Composition	ε_r_	Q × f (GHz)	τ_f_ (ppm/°C)	Reference
0.925ZnNb_2_O_6_-0.075Ca_0.5_Sr_0.5_TiO_3_	23.39	21,686	−8.99	This study
0.5ZnNb_2_O_6_-0.5CoTiNb_2_O_8_	39.2	40,013	3.57	[29]
ZnNb_2_O_6_-8.75 wt.%SrTiO_3_	24.6	48,230	0	[44]
ZnNb_2_O_6_-Zn_0.9_Mg_0.1_TiO_3_	27.5	75,000	3.8	[45]
0.8ZnTiNb_2_O_8_-0.2CuTiNb_2_O_8_	35.01	19,449	−0.3	[46]
ZnTiNb_2_O_8_ (ZnNb_2_O_6_-TiO_2_)	35.5	52,500	−60	[47]
0.4ZnNb_1.98_(TiW)_0.02_O_6_-0.6Ni_0.5_Ti_0.5_NbO_4_	31.7	29,800	0	[48]

## Data Availability

The original contributions presented in this study are included in the article. Further inquiries can be directed to the corresponding author.
